# Characterization of Exosomal SLC22A5 (OCTN2) carnitine transporter

**DOI:** 10.1038/s41598-018-22170-7

**Published:** 2018-02-28

**Authors:** Lara Console, Mariafrancesca Scalise, Annamaria Tonazzi, Nicola Giangregorio, Cesare Indiveri

**Affiliations:** 1 Department DiBEST (Biologia, Ecologia, Scienze della Terra) Unit of Biochemistry and Molecular Biotechnology, University of Calabria, Via Bucci 4C, 87036 Arcavacata di Rende, Italy; 2 CNR Institute of Biomembranes and Bioenergetics, via Amendola 165/A, 70126 Bari, Italy

## Abstract

Exosomes are extracellular vesicles involved in cell-to-cell communication. Previous large scale proteomics revealed that they contain SLC proteins. However, no data on the function of exosomal SLCs is available, so far. An SLC localized in exosomes was here characterized for the first time: the carnitine transporter OCTN2 (SLC22A5). The protein was detected by Western Blot analysis in HEK293 exosomes. To investigate the functional properties of the exosomal OCTN2, the proteins extracted from vesicles were reconstituted into proteolipsomes and the transport function was measured as uptake of ^3^H-carnitine. Transport was stimulated by sodium and was dependent on pH. ^3^H-carnitine uptake was inhibited by Acetyl-carnitine, but not by Asn, Gln and Arg thus excluding interference by ATB^0,+^, an amino acid transporter which also recognizes carnitine. Cardiolipin failed to stimulate transport, excluding the activity of the mitochondrial Carnitine/acylcarnitine transporter. Increased level of exosomal OCTN2 was induced by treatment of HEK293 with the pro-inflammatory cytokine INFγ. All data concurred to demonstrate that OCTN2 present in exosomes is fully functional and is in its native conformation. Functional OCTN2 was detected also in human urinary exosomes, thus suggesting the OCTN2 exosomal protein as a candidate biomarker for inflammation related pathologies.

## Introduction

Several studies showed that eukaryotic cells release different types of vesicles into the extracellular environment among which exosomes represent a sub set with definite physicochemical and biochemical characteristics. They are 20–100 nm in size with a density of about 1.10 g × ml^−1^ and are enriched in specific proteins such as TGS101 and CD9, which are acknowledged as exosomal markers^1,2^. A growing number of evidences suggest that exosomes play an important role in cell-to-cell communication under both physiological and pathological conditions. Indeed, exosomes are involved in immune response, angiogenesis, inflammation, cell death, neurodegenerative diseases and cancer malignancy^3^. Knowledge of exosomal cargo composition opens new perspectives in the field of diagnostics. Indeed, exosomes collected from body fluids such as blood, urine, ascites, cerebrospinal fluid and milk, could be used as biomarkers of the tissues from which they originate. Large-scale proteomics have been previously performed to search for exosomal molecular cargos. Interestingly, these studies revealed the presence of several proteins belonging to SLC families^4^, which include over 400 membrane transporters that are fundamental players in cell metabolisms^5^. Despite the important role of the transporters in cell life, the physiological significance of the presence of this class of protein in exosomes is unknown. Indeed, apart from the proteomic data, few information is available about the function of SLCs in this exocellular location. In this study, the first characterization of an SLC member present in exosomes is described. SLC22A5, also known as OCTN2, is a sodium dependent carnitine transporter belonging to the sub-family of Organic Cation Transporters Novel and widely expressed in human tissues^6,7^. In cells, OCTN2 plays a crucial role in the cellular uptake of L-carnitine, which is an essential co-factor for the mitochondrial β-oxidation pathway^8^. Indeed, humans can synthesize only a relatively small fraction of the required L-carnitine that, hence, has to be absorbed from diet^9^. For this reason, OCTN2 is essential for completion of the β-oxidation pathway as demonstrated by occurrence of a severe disease, known as primary carnitine deficiency^10^, caused by OCTN2 gene defects. The pathology is characterized by impaired intestinal and muscular uptake and increased renal loss of carnitine^9^. Recent studies demonstrated that expression level of OCTN2 is altered in many human cancers^11^. This alteration, which is induced by DNA methylation^12^, may cause impairing of the mitochondrial β-oxidation in cancer growth and progression. This serves for switching the cancer cells from lipid to glycolytic metabolism according to Warburg effect^13^. Very interestingly, OCTN2 is implicated in the inflammatory response. Its gene is located on the “Inflammatory Bowel Diseases 5” risk region (IBD). Moreover, Mikihiro Fujiya *et al*.^14^ demonstrated that epithelial OCTN2 expression is increased by inflammation, through increased levels of pro-inflammatory cytokines such as INF-γ. Therefore, investigation of OCTN2 association with exosomes was of interest for human physio/pathology. OCTN2 and exosomes share similar links to human pathology, thus increasing the interest in characterizing the transporter in this alternative location.

## Results

### Exosomes harbor functional OCTN2

**Figure 1:**
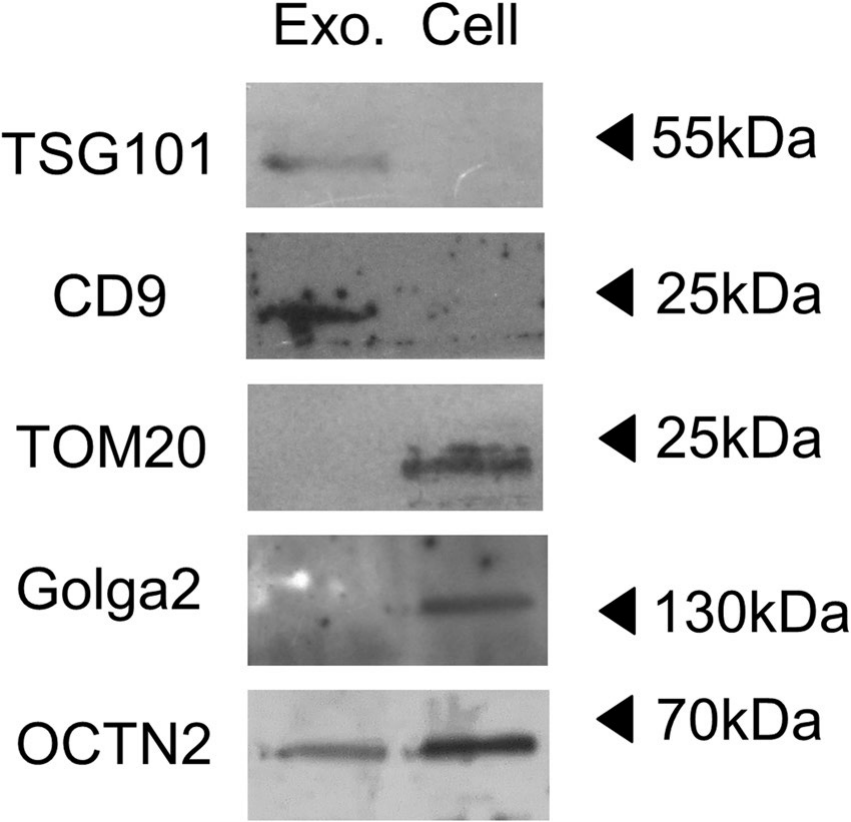
Immunoblot analysis of exosomes isolated from HEK293. Proteins from exosomes (left line) or cell extract (right line) were separated on SDS-PAGE gels and blotted. Then, the membrane was cut for incubation with the antibodies against TSG101, CD9 (conventional exosomal marker), TOMM20, Golga2 (mitochondrial and Golgi marker respectively) and OCTN2. The Molecular Mass of standard proteins (Thermo Scientific PageRuler Plus Prestained Protein Ladder) is indicated by arrows. The image is representative of three independent experiments.

To detect the presence of OCTN2 in exosomes and to gain further insights into the possible role of the transporter, exosomes from HEK293 were isolated according to the well assessed method of ultracentrifugation^15^. The enrichment of exosomes in the preparation was confirmed by WB analysis using typical exosome markers that is CD9 and TSG101. Figure 1 shows immunodetection of the two markers in the exosome preparation, while no or very few reaction was observed in the cell extract. This data confirmed that the isolated vesicles were, indeed, exosomes. The purity of the preparation was assessed by Anti-Tom 20 and Anti-Golga2, that is antibodies against specific markers of mitochondria and Golgi, respectively^16,17^. In this case, the absence of immunostaining in the exosomal extract allowed us to exclude contaminations by mitochondria and Golgi. As expected, positive reaction by Anti-Tom 20 and Anti-Golga2 was observed in the cell extract (Fig. 1). Then, the exosome fraction, was used to perform WB analysis with the Anti-OCTN2 antibody. A band was detected confirming the presence of OCTN2 in exosomes isolated from HEK293 medium. The transporter was also detected in the cell extract, in line with its native location in the plasma membrane (Fig. 1). By comparison with standard proteins, the apparent molecular mass of OCTN2 was about 70 kDa in both the exosomal and the cell fractions. The specificity of the antibody for OCTN2 was assessed as shown in Supplementary Fig. 1.

**Figure 2:**
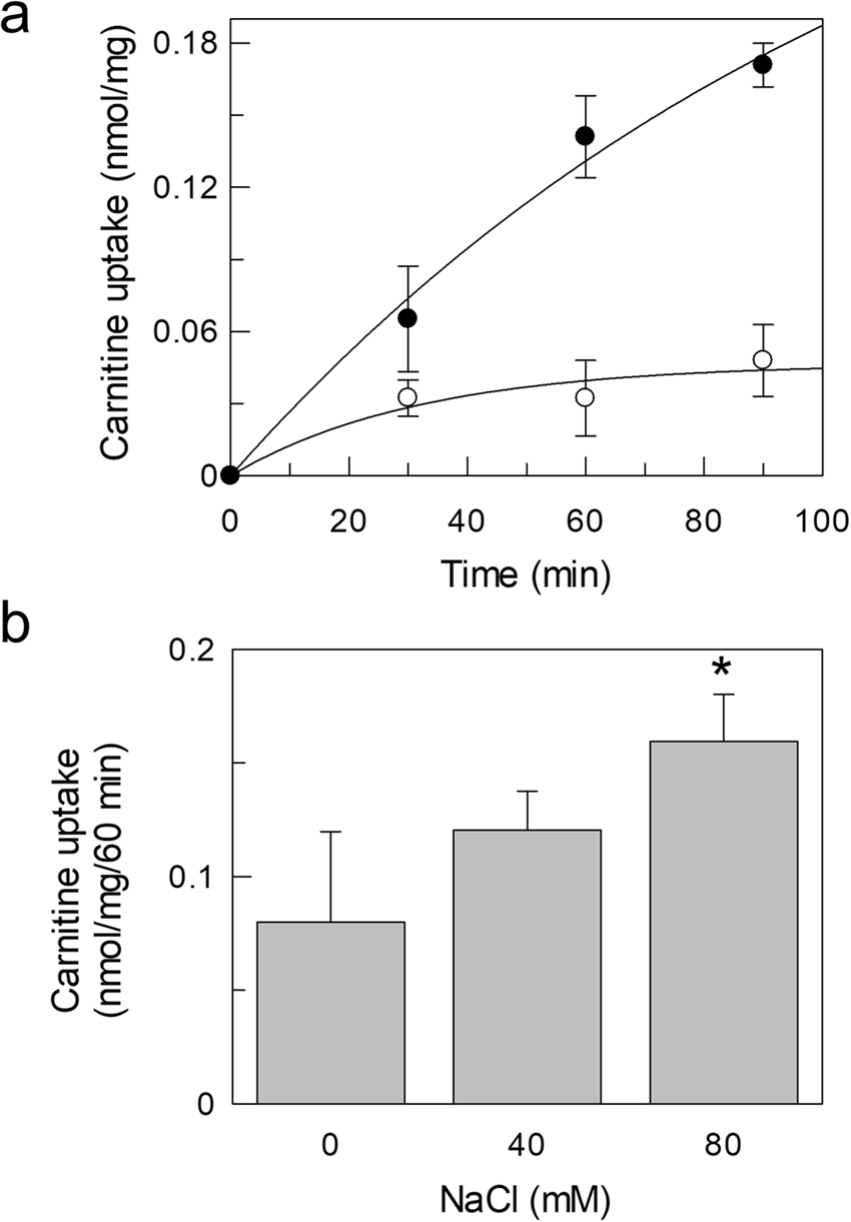
Sodium dependent carnitine uptake in proteoliposomes by exosomal OCTN2. Proteoliposomes were obtained by reconstitution of exosomal protein extract as described in Materials and Methods. (**a**) Transport activity was started adding 50 µM ^3^H-carnitine to proteoliposomes and terminated at the indicated times loading the samples on cold columns containing Sephdex G-75. The uptake was measured in presence (⦁) or absence (○) of external 80 mM NaCl. (**b**) NaCl at the indicated concentrations was added to proteoliposomes. After 2 min of incubation, carnitine transport was started adding 50 µM ^3^H-carnitine and stopped after 60 min. The osmolarity of each sample was balanced by substituting the NaCl with an appropriate concentration of sucrose. The values are the mean ± SD from three experiments. (*) Significantly different as estimated by the Student’s t test (p < 0.05).

**Figure 3:**
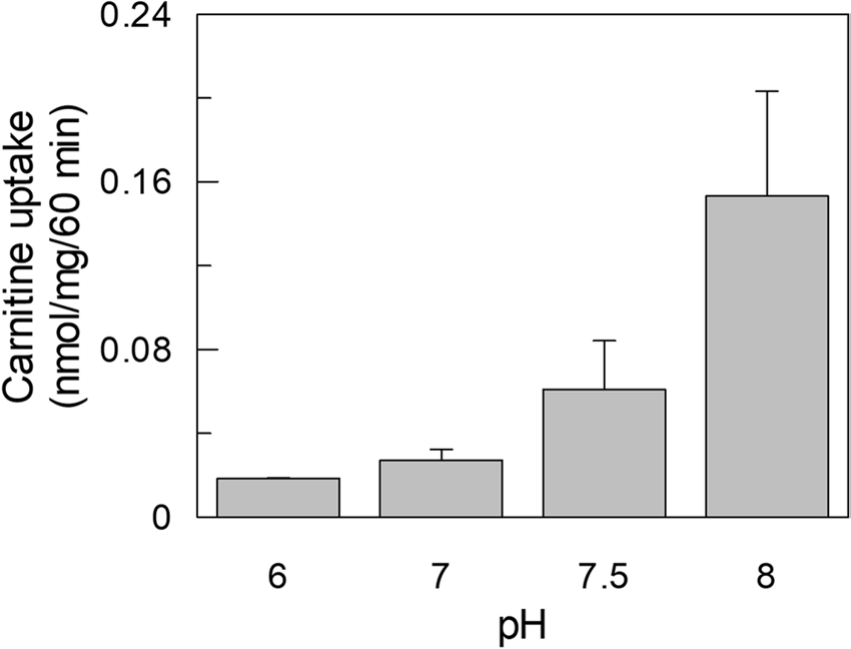
pH dependence of carnitine uptake in proteoliposomes. Proteoliposomes were obtained by reconstitution of exosomal protein extract at the indicated pH, as described in Materials and Methods. Transport activity was started adding 50 µM ^3^H-carnitine to proteoliposomes and terminated at 60 min as in Fig. 2. The values are the mean ± S.D. from three independent experiments.

**Figure 4:**
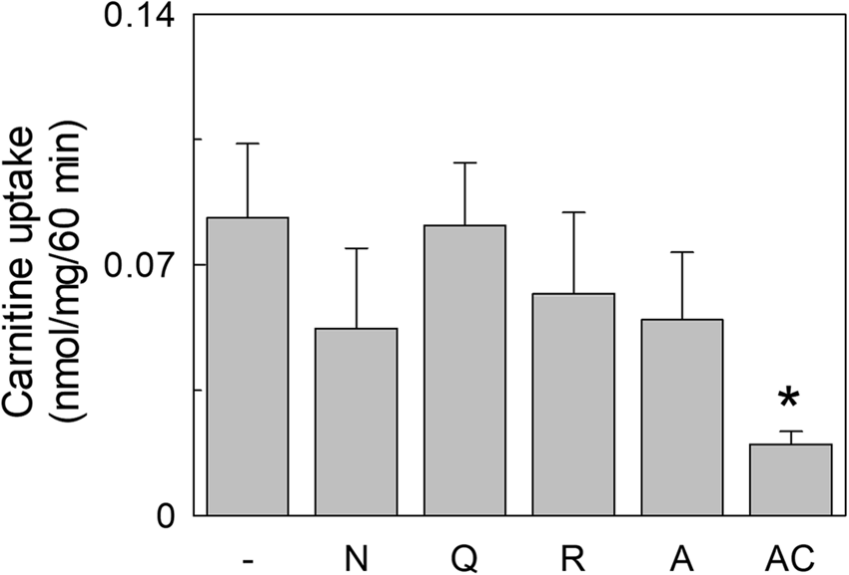
Inhibition of carnitine uptake by amino acids and acetylcarnitine. 2.5 mM Asn, Gln, Arg, Ala or Acetyl-carnitine were added to proteoliposomes, obtained as in Fig. 2, together with 10 µM ^3^H-carnitine. Transport was stopped after 60 as in Fig. 2. The data represent means ± S.D. of three independent experiments. (*) Significantly different as estimated by the Student’s t test (p < 0.05).

To ascertain whether the transporter present in exosome was functional, HEK293-derived vesicles were solubilized with Triton X-100 and reconstituted into proteoliposomes. The transport function was measured as uptake of ^3^H-carnitine. The time course in Fig. 2a shows accumulation of labeled carnitine in proteoliposomes which was stimulated by externally added NaCl. Indeed, activity was dependent on NaCl concentration (Fig. 2b). NaCl concentration higher than 80 mM did not further stimulate transport (Supplementary Fig. 2a). Similar results were observed when using 1 µM carnitine instead of 50 µM (Supplementary Fig. 2b). Na-gluconate exerted the same effect (not shown) thus excluding the influence of Cl^−^ on transport. It should be noted that a small amount of Sodium (about 10 mM) is present in the buffer used for reconstitution and transport, since it cannot be substituted by TRIS or Potassium which negatively affect transport. Therefore the actual stimulation by Sodium is higher than that observed. This finding suggests that the carnitine uptake into proteoliposomes was mediated by a functional OCTN2. To definitively assess the nature of the transporter, some other functional properties were tested. Exosome protein extract was reconstituted using buffers at different pH. As shown in Fig. 3 the carnitine uptake increased from pH 6.0 to pH 8.0. The same behavior was found in HEK293 extract, reconstituted in proteoliposomes (not shown) and, previously, in intact cells. Indeed, this is a typical feature of Organic Cation Transporters^18^. Furthermore, the inhibitory effect of Acetyl-carnitine and Asn, Gln, Arg and Ala on carnitine transport was tested in order to exclude that the carnitine uptake was due to ATB^0,+^ (SLC6A14), an amino acids transporter which also recognizes carnitine (Fig. 4). Acetyl-carnitine inhibited the transport activity by about 75%, while the amino acids showed poorly or not significant inhibition. This behavior is completely different from the inhibition pattern previously described for ATB^0,+^^19^. This confirms that the observed transport function can be ascribed to OCTN2.

**Figure 5:**
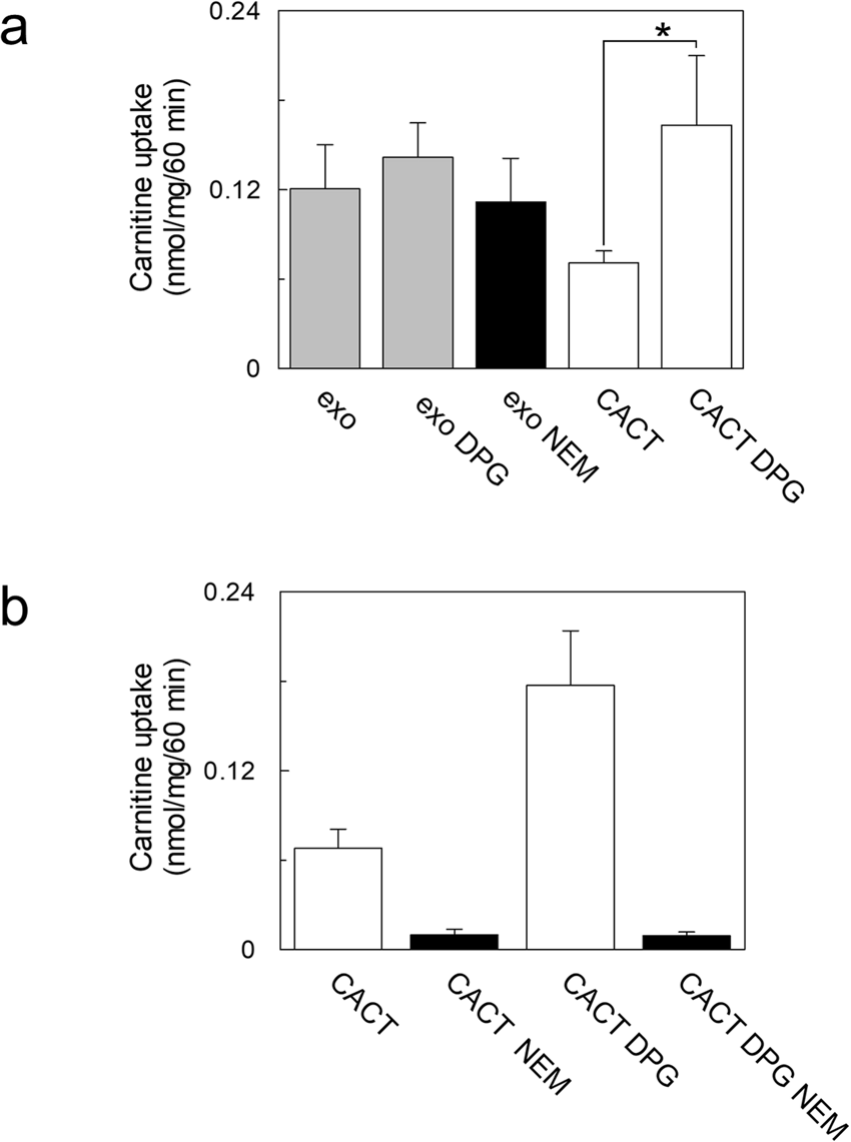
Effect of cardiolipin (DPG) and NEM on carnitine uptake in proteoliposomes. (**a**) Exosomal extract (grey bar) or mitochondrial extract (white bar) was reconstituted in proteoliposomes with or without cardiolipin (see Materials and Methods). The black bar shows the inhibition by 0.1 mM NEM of carnitine uptake into proteoliposome reconstituted with exosomal extract. (**b**) Mitochondrial extract (white bars) was reconstituted in proteoliposomes with or without cardiolipin. The black bars show the inhibition by 0.1 mM NEM of carnitine uptake into proteoliposome with or without cardiolipin. Transport was started adding 50 µM ^3^H-carnitine to proteoliposomes and stopped after 60 min. The values are the mean ± S.D. from three independent experiments. (*) Significantly different as estimated by the Student’s t test (p < 0.05).

To exclude that the carnitine uptake could be due in part to the presence of contamination by the mitochondrial carnitine carrier (CACT), the dependence on cardiolipin (DPG), which specifically stimulates the activity of the mitochondrial transporter^20^, was checked. To this aim, cardiolipin was included into the proteoliposome membrane as previously reported^21^. No effect was observed upon addition of cardiolipin, excluding the presence of the mitochondrial transporter (Fig. 5). To further substantiate the absence of CACT contaminations, the inhibition of carnitine uptake by N-Ethylmaleimide (NEM), a strong inhibitor of the mitochondrial transporter, was tested. The response to NEM was very poor compared with the well described inhibition of the mitochondrial transporter (Fig. 5b)^22^. As a control, cardiolipin was included into proteoliposomes reconstituted with HEK293 mitochondria extract and the CACT activity assayed. As expected, a clear activation was observed (Fig. 5). Taken together, the data confirms the presence of functional OCTN2 into HEK 293 derived exosomes.

### Increase of OCTN2 level in exosomes

**Figure 6:**
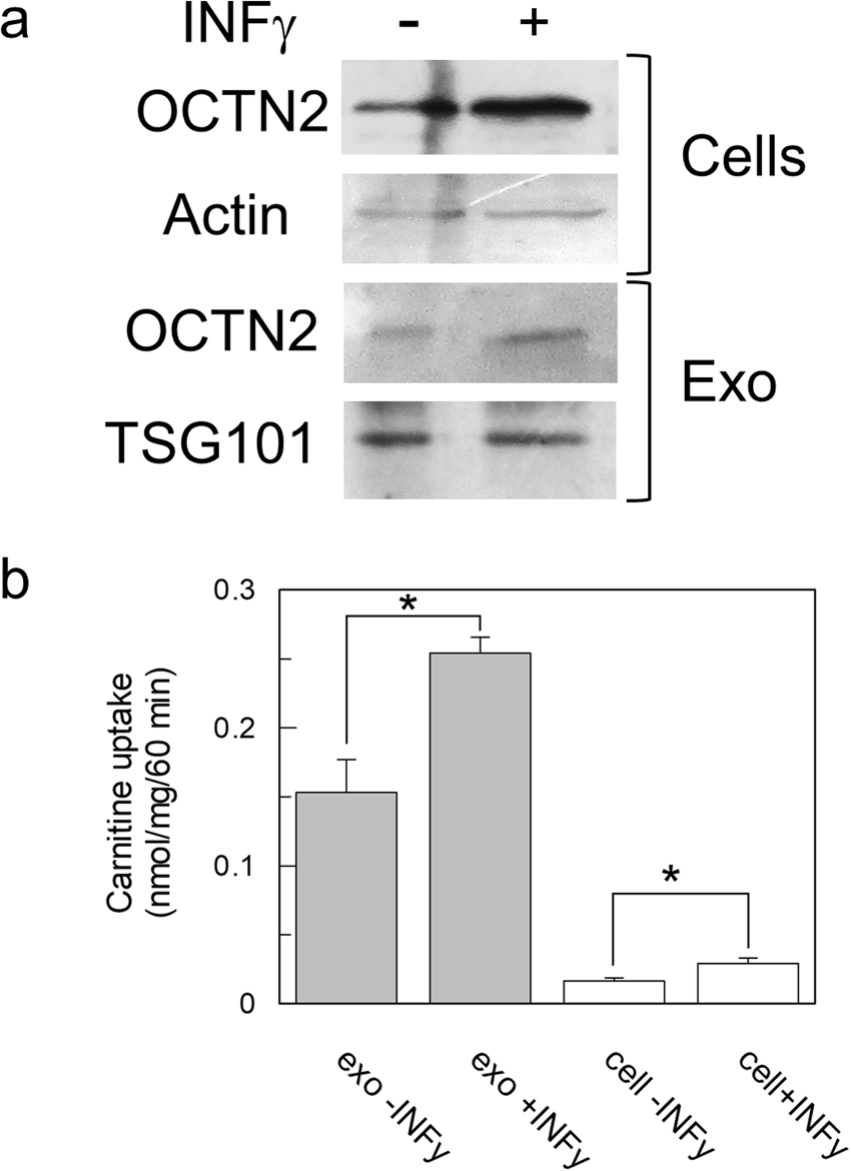
Effect of IFN-γ on OCTN2 level in cell and exosomes. HEK293 cells were treated with 50ng/ml IFN-γ for 48 hours. After incubation, cells were harvested. Exosomes were isolated from the medium of the same culture. (**a**) Both cell (upper panel) and exosomal (lower panel) protein extracts deriving from IFN-γ treated (right line) or untreated (left line) culture was analyzed by WB using the antibody against OCTN2. The immunostaining of cell extract with the antibody against actin was used as loading control. Exosomal OCTN2 was normalized using TSG101 by loading on SDS-PAGE an equal amount of the same exosomal extracts used to test the OCTN2 expression. Cell lysate and exosomal extract were subjected to WB. The membrane was cut for incubation with the different antibodies (**b**) Transport was started adding 50 µM ^3^H-carnitine to proteoliposomes and stopped after 60 min. Proteoliposome was reconstituted using either exosomal (gray bar) or cell (white bar) protein extracts. The values are the mean ± S.D. from three independent experiments. (*) Significantly different as estimated by the Student’s t test (p < 0.05).

To gain some insights in the possible mechanism of OCTN2 insertion in exosomes, the expression of the transporter in HEK293 cells was increased by Interferon-γ (INFγ) treatment, as previously reported^14^. Indeed, incubation of HEK293 cells with INFγ for 48 h led to increase of OCTN2 expression in the cells, as evidenced by WB analysis (Fig. 6a). Exosomes deriving from treated cells also contained higher amount of OCTN2 (Fig. 6a). The activity of the OCTN2 extracted from the exosome derived from the medium of treated or untreated cells was tested in reconstituted proteoliposomes to confirm the data obtained by WB. An increase of activity of about 40% was found after treatment with INFγ (Fig. 6b). The activity of OCTN2 extracted from cells, not treated or treated with INFγ, was also measured. An increased transport activity was observed which correlated well with the increased expression.

### OCTN2 in urinary exosomes

**Figure 7:**
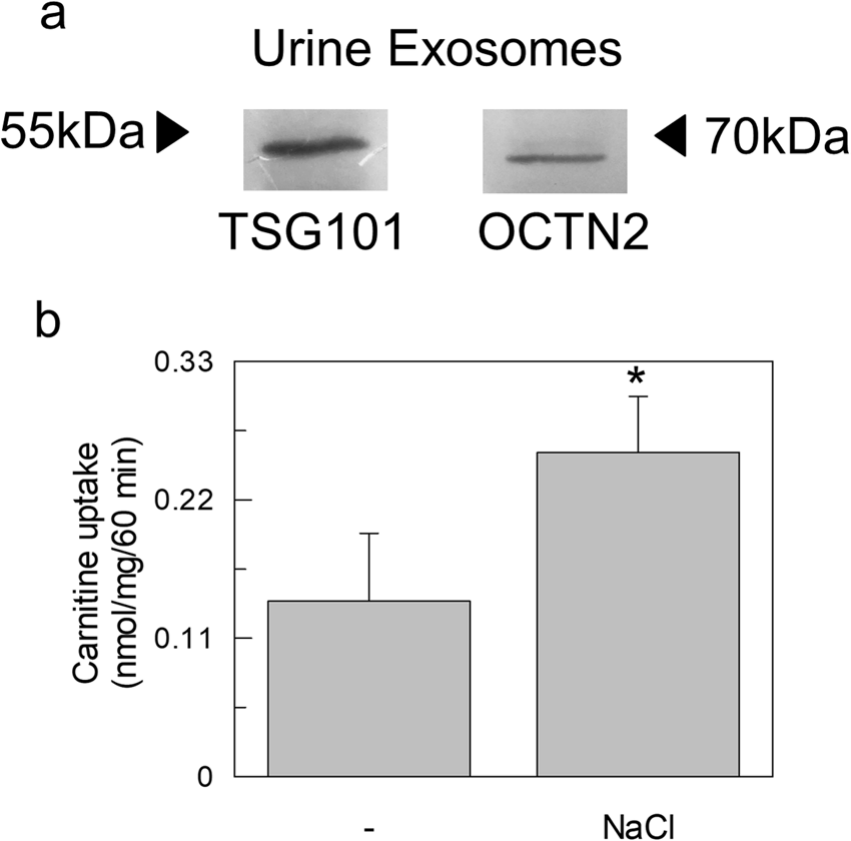
OCTN2 in human urine exosomes. (**a**) Proteins from human urine derived exosomes were separated on SDS-PAGE gels, and blotted. Then, the membrane was cut for incubation with the antibodies against TSG101and OCTN2. The Molecular Mass of standard proteins (Thermo Scientific PageRuler Plus Prestained Protein Ladder) is indicated by arrows. (**b**) Transport activity was started adding 50 µM ^3^H-carnitine to proteoliposomes and stopped after 60 min as in Fig. 2. The uptake was measured in presence or absence of external 80 mM NaCl. The values are the mean ± SD from three experiments. (*) Significantly different as estimated by the Student’s t test (p < 0.05).

To characterize OCTN2 in urinary exosomes, the vesicles were extracted from human urine. In WB analysis, immunostaining of OCTN2 was observed in these vesicles (Fig. 7a). To ascertain the functionality of the protein, the uptake in reconstituted proteoliposomes was detected in the absence or presence of 80 mM NaCl (Fig. 7b). Uptake was stimulated by Na^+^ confirming the presence of functional OCTN2 also in human urine exosomes.

## Discussion

Initial studies showed that eukaryotic vesicles are used to remove obsolete cellular components. More recently, the hypothesis that exosomes play a pivotal role in intercellular communication by carrying functional proteins and nucleic acids has been considered^3^. However, molecular and functional characterization of cargos is still poor especially to what concerns membrane transporters. The interest in studying exosomal OCTN2 derives from its crucial role in cell homeostasis and from the association of transporter defects to human diseases, such as inflammatory diseases, including IBD. This is even more relevant in the frame of the findings that also exosomes have been linked to IBD etiology^23,24^. Previous proteomic studies revealed the presence of amino acid sequences belonging to OCTN2, among others SLC, in human urine exosomes^25^. In line with this, the results here described highlight that exosomes collected from human urine contain functional OCTN2. This correlates well with the high expression level of OCTN2 in kidney, besides other tissues^18^. Investigating SLC members could help to determine the cellular origin of urine exosomes. To characterize in more details the human exosomal OCTN2, we have exploited HEK293 cells. It was reported that these immortalized cells maintain some features of renal cells and are able to release exosomes^26,27^, even though, they show also some properties of neuronal cells. Noteworthy, exosomes deriving from a human kidney cell line are more appropriate to study OCTN2 compared to exosomes collected from urine of animal model such as rat or mouse. Indeed, these animals harbor different patterns of carnitine transport and metabolism with respect to humans. As an example, murine genome encodes for the third carnitine transporter (OCTN3)^28^, which is not present in humans^7^. Biochemical characterization of OCTN2 suggested that the protein in exosomes, derived from HEK293 cells, is in a glycosylation state equal to that of native plasma membrane. This is indicated by the apparent molecular mass of 70 kDa (see Fig. 1 and Fig. 7) which is much higher than the theoretical mass of the protein or than the apparent molecular mass of the OCTN2 expressed in bacteria that cannot assemble glycosylation moieties (see Supplementary Fig. 1)^29^. The glycosylation state of OCTN2 suggests a correct folding in the exosomal membrane that was indeed confirmed by the activity assay. Thanks to the proteoliposome approach, the protein extracted from both cells and exosomes could be assayed. It is worth noting that the transport activity of exosomal OCTN2 is enriched if compared with the cellular one. This probably depends on the fact that the exosomal extract contains less total proteins than the cell extract. Indeed, in some previous studies, the activity of OCTN2 was detectable in intact cells only after the induction of the overexpression of the transporter by transfection^18^. The measured carnitine uptake using exosomal extracts showed all the main features of OCTN2 located at the plasma membrane^18^. Importantly, interferences by other transporters on the observed carnitine uptake activity was excluded. In particular, the mitochondrial carnitine carrier could be excluded by the lack of cardiolipin dependence and NEM sensitivity^20^. Another transporter which was shown to exhibit specificity for carnitine is ATB^0,+^, whose main function consists in mediating amino acid transport. Its presence could be excluded by absence of specificity towards amino acids transported by ATB^0,+^ (see Fig. 4). OCTN2 expression in exosomes is regulated by one of the same factors that regulates its expression in cells, i.e., the pro-inflammatory cytokine INFγ. This suggests that the abundance of OCTN2 in exosomes relates to expression in cells. This finding may suggest a relevance of the exosomal OCTN2 in pathological contexts such as inflammation.

Several studies showed that OCTN2, its substrate carnitine and exosomes are involved in inflammatory process. On the one hand, carnitine has been associated to inflammation related-diseases including diabetes mellitus^30^, chronic renal failure, cardiomyopathy, cirrhosis and sepsis^8^. Indeed, carnitine administration to patient in hemodialysis significantly reduces serum C-Reactive Protein (CRP) and Serum Amyloid A (SAA), two systemic inflammation markers^31^. On the other hand, OCTN2 is associated with Crohn’s disease and ulcerative colitis^32–34^. The intestine of neonatal homozygous mouse, in which the OCTN2 is deleted, displayed early signs of inflammation, such as lymphocytic and macrophage infiltration. This is in line with the ability of OCTN2 to mediate the absorption of the pentapeptide Competence and Sporulation Factor (CSF) into the intestinal cells. This factor induces cytoprotective mechanisms, which prevent intestinal epithelial cell injury and loss of barrier function^35^. Therefore, Shtrichman *et al*. showed that INFγ, which plays a crucial role in the defense against microbial infections^36^, increase the OCTN2 expression. This suggests that OCTN2 may participate to restoration of intestinal homeostasis under inflammation stress. Moreover, the protein composition of exosomes can be influenced by pro-inflammatory cytokines as demonstrated in the case of neutral ceramidase (NCDase) whose expression is reduced in exosomes in the presence of high level of pro-inflammatory cytokines and vice versa^37^. The described results represent an encouraging starting point to further investigate the link between exosomal OCTN2 and inflammatory diseases with important implications in diagnosis.

## Methods

### Materials

Sephadex G-75 was purchased from Pharmacia, l-[methyl-3H]carnitine from Amersham, egg-yolk phospholipids (l-α-phosphatidylcholine from fresh turkey egg yolk), Ethylenedinitrilo tetraacetic acid (EDTA), 4-(2-Hydroxyethyl)piperazine-1-ethanesulfonic acid (HEPES), 4-(1,1,3,3-Tetramethylbutyl)phenyl-polyethylene (Triton X-100), cardiolipin (DPG) and L-carnitine from Sigma, St. Louis, MO. Human embryonic kidney HEK293 cells were obtained from the American Type Culture Collection (ATCC). Tissue culture media and fetal bovine serum were purchased from Life Technologies. INFγ, SLC22A5 and CD9 Antibodies were purchased from Thermo Fisher Scientific, TSG101, TOMM20 and Golga2 from Santa Cruz Biotechnology.

### Cell culture

HEK 293 cells were maintained in Dulbecco’s Modified Eagle Medium (DMEM) supplemented with 10% (v/v) fetal bovine serum (FBS), 1 mM glutamine, 1 mM sodium pyruvate and Pen/Strep as antibiotics. Cells were grown on 10 cm^2^ plates at 37 °C in a humidified incubator and a 5% CO_2_ atmosphere. In case of collection of exosome, conditioned medium was used in place of classical one.

### Cytokine treatment

HEK 293 cells were cultured using standard culturing conditions seeded onto 12 well plates up to 50–70% confluence. 50 ng/ml of IFNγ was added for 48 h in a serum free medium according to manufacturer’s conditions.

### Exosome isolation

Urine (from a human individual) or culture media was centrifuged at 15,000 g for 10 min to pellet cells and other debris. The supernatant was then centrifuged at 150,000 g for 120 min to pellet the exosomal fraction. The pellet was washed with phosphate-buffered saline (PBS) and then re-centrifuged at 150,000 g for 120 min before final resuspension^15^. The protocol regarding the collection of human urine was carried out in accordance to the relevant guidelines and regulations. All experimental protocols were approved by the Institutional Ethic Committee of the University of Calabria. The informed consent was obtained from all healthy volunteer subjects.

### Exosome and cell solubilisation

Exosomal fraction were resuspended in the lysis buffer made of 3% Triton X-100 in 20 mM Hepes/NaOH pH 7.5 and protease inhibitors. After incubation in ice for 30 min the lysate was centrifuged at 12,000 g for 15 min at 4 °C. The supernatant was used for further analysis. The same procedure and buffer were used to solubilized HEK293 cells pellets which were harvested at the same time of media collection.

### Mitochondria isolation from HEK293

HEK293 cells were harvested and resuspended in cold Isolation Buffer (10 mM HEPES, pH 7.4, 300 mM mannitol and 0.2 mM EDTA). After that, cells were homogenized on ice with a 2-ml glass potter and mitochondria was isolated using a differential centrifugation method^38^.

### SDS-PAGE and western blot analysis

SDS-PAGE electrophoresis was performed in the presence of 0.1% SDS according to Laemmli. Stacking gel and separation gel were prepared as 4% and 12% acrylamide respectively (acrylamide/bisacrylamide ratio 30:0.2). Proteins were transferred to nitrocellulose membrane. The membrane was immunostained using anti-OCTN2, anti-CD9, anti-TSG101, anti-Golga2 and anti-TOMM20 antibodies in accordance with the directions manufacturer. The WB images are representative of at least two independent experiments.

### Reconstitution in proteoliposomes

Total protein extract from exosomes or HEK293 cell lysate was reconstituted by removing the detergent from mixed micelles containing detergent, protein and phospholipids. The composition of the initial mixture was: 200 μg of total proteins, 75 μl of 10% Triton X-100, 110 μl of 10% egg yolk phospholipids in the form of sonicated liposomes 20 mM Hepes (pH 7.5) in a final volume of 700 μl.

### Transport measurements

550 μl of proteoliposomes were passed through a Sephadex G-75 column. The first 600 μl of the turbid eluate from the Sephadex column were collected, transferred to reaction vessels (100 μl each), and readily used for transport measurement. Transport was performed at 25 °C and started by adding 0.05 mM ^3^H-carnitine to proteoliposomes. Transport assay was terminated at the required time interval. Finally, the external substrate was removed by chromatography on Sephadex G-75 columns, and the radioactivity in the liposomes was measured.

### Statistical test

A two tailed t-tests was performed to calculate the significant difference between samples.

## Electronic supplementary material

Supplementary information
