# Supplementary material for: Characterization of Exosomal SLC22A5 (OCTN2) carnitine transporter
Source: Sci Rep. 2018 Feb 28;8:3758. doi: 10.1038/s41598-018-22170-7 (PMC5830701; doi:10.1038/s41598-018-22170-7)
Supplement: Supplementary file 1 — Supplementary information [file 41598_2018_22170_MOESM1_ESM.pdf]

Characterization of Exosomal SLC22A5 (OCTN2) carnitine transporter.

Lara Console[1], Mariafrancesca Scalise[1], Annamaria Tonazzi[2], Nicola Giangregorio[2], Cesare Indiveri[1, 2*]

[1] Department DiBEST (Biologia, Ecologia, Scienze della Terra) Unit of Biochemistry and Molecular Biotechnology, University of Calabria, Via Bucci 4C, 87036 Arcavacata di Rende, Italy

[2] CNR Institute of Biomembranes and Bioenergetics, via Amendola 165/A, 70126 Bari, Italy

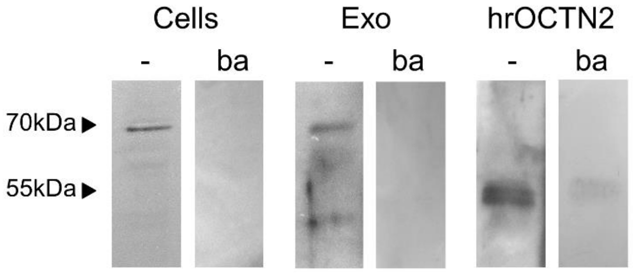

Fig. S1 Specificity of the anti-OCTN2 antibody. A WB experiment using the recombinant human OCTN2 as a blocking peptide was performed. 20 µg of cell extract (Cells), 20 µg of exosomal extract (Exo) and 2 µg of purified recombinant human OCTN2 (hrOCTN2) were run on SDS-PAGE and blotted on nitrocellulose membrane for immune-detection by anti-OCTN2. Before proceeding with the staining, 5 µg of anti-OCTN2 was incubated with 50 µg of the purified hrOCTN2. The blocked anti-OCTN2 antibody (ba) was used for staining in parallel with the untreated antibody (-). The absence or presence of faint bands in the lines immuno-stained with the blocked anti-OCTN2 (ba) demonstrates specificity of the anti-OCTN2 antibody. The image is representative of two independent experiments.

a

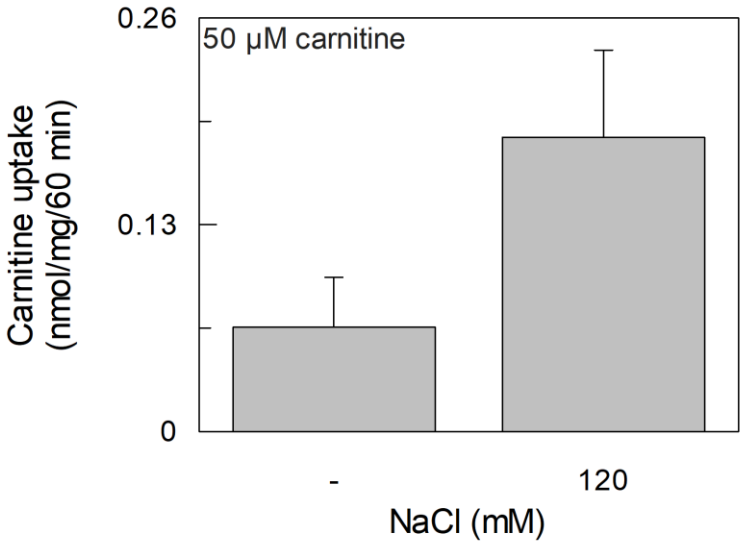

b

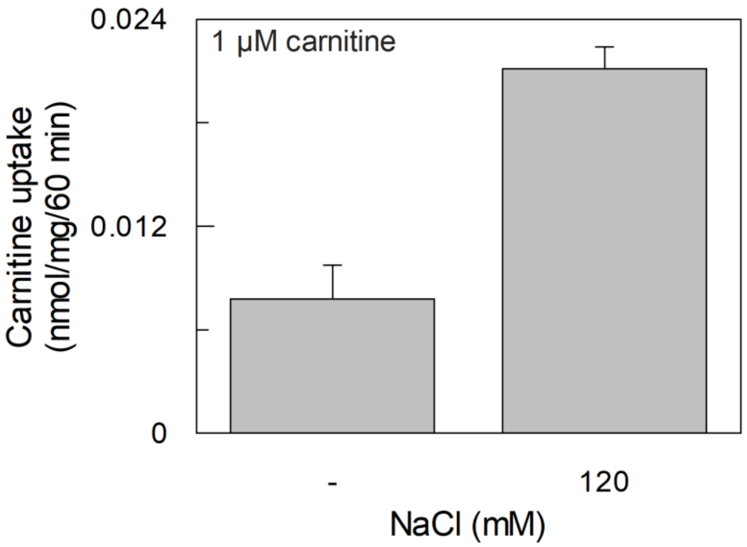

Fig. S2. Effect of Sodium on carnitine uptake in proteoliposomes by exosomal OCTN2. OCTN2 reconstitution into proteoliposomes was performed removing the detergent from mixed micelles obtained mixing 200 µg of total proteins from exosome,75 µl of 10% Triton X-100, 110 µl of 10% sonicated liposomes 70 µl of Hepes 200mM at pH 7.5 in a final volume of 700 µl. The proteoliposomes were passed through a Sephadex G-75 column and the eluate was used for transport measurement. Transport was started by adding 0.05 mM ^{3}H-carnitine to proteoliposomes (a) or 0.001 mM ^{3}H-carnitine (b) and termed by removing the external substrate. The concentrations of ^{3}H-carnitine used in a and b are lower to the Km of SLC6A14 and SLC22A4 which are low affinity transporters of carnitine to minimize their contribution to the measured transport activity. In both cases the stimulus of the transport activity by 120mM NaCl, a concentration which is in the physiological range, was evaluated. The values are the mean ± SD from three experiments.
